# Cytogenetics, cytotaxonomy and chromosomal evolution of Chrysomelinae revisited (Coleoptera, Chrysomelidae) *

**DOI:** 10.3897/zookeys.157.1339

**Published:** 2011-12-21

**Authors:** Eduard Petitpierre

**Affiliations:** 1 Dept. of Biology, University of Balearic Islands, 07122 Palma de Mallorca, Spain

**Keywords:** chromosomes, leaf-beetles, diploid numbers, modal numbers, sex-chromosome systems, intrageneric evolution, karyotype symmetry

## Abstract

Nearly 260 taxa and chromosomal races of subfamily Chrysomelinae have been chromosomally analyzed showing a wide range of diploid numbers from 2n = 12 to 2n = 50, and four types of male sex-chromosome systems. with the parachute-like ones Xy_p_ and XY_p_ clearly prevailing (79.0%), but with the XO well represented too (19.75%). The modal haploid number for chrysomelines is n = 12 (34.2%) although it is not probably the presumed most plesiomorph for the whole subfamily, because in tribe Timarchini the modal number is n = 10 (53.6%) and in subtribe Chrysomelina n = 17 (65.7%). Some well sampled genera, such as *Timarcha*, *Chrysolina* and *Cyrtonus*, are variable in diploid numbers, whereas others, like *Chrysomela*, *Paropsisterna*, *Oreina* and *Leptinotarsa*, are conservative and these differences are discussed. The main shifts in the chromosomal evolution of Chrysomelinae seems to be centric fissions and pericentric inversions but other changes as centric fusions are also clearly demonstrated. The biarmed chromosome shape is the prevalent condition, as found in most Coleoptera, although a fair number of species hold a few uniarmed chromosomes at least. A significant negative correlation between the haploid numbers and the asymmetry in size of karyotypes (r = -0.74) has been found from a large sample of 63 checked species of ten different genera. Therefore, the increases in haploid number are generally associated with a higher karyotype symmetry.

## Introduction

The subfamily Chrysomelinae is a large cosmopolitan taxon of nearly 2000 species (Farrell 1998) or even 3000 worldwide species (Reid et al. 2009), in some 133 genera (Daccordi 1994). They are mostly round and highly convex leaf-beetles, living mainly in temperate regions of Australia and South America, but well represented also in the Holarctic region (Daccordi 1982). A very interesting feature of the species in this subfamily is a striking ecological specialisation, due to their trophic selection on plants usually belonging to the same botanic family, and very often even, on one or a few closely related plant genera (Jolivet and Hawkeswood 1995). Chrysomelinae has been characterized as a fixed taxonomic group which can be distinguished by many apomorphic characters of adults and larvae (Chen 1935). Recent molecular and morphological studies support their monophyletic origin (Duckett et al. 2004; Farrell and Sequeira 2004), although a much larger sampling on 30 species indicates paraphyly of *Timarcha* with regard to the other chrysomelines (Gómez-Zurita et al. 2008).

The current cytogenetic findings in this subfamily cover a total of 259 taxa and chromosomal races, that is between 8,6% and 13.0% of those described, which have been surveyed at least with the first level of chromosomal knowledge, usually called α-karyology (White 1978), basically referred to diploid numbers and sex-chromosome systems. Since our first published list of karyologically checked taxa of Chrysomelinae along with the remaining leaf beetles (Petitpierre et al. 1988), and their rough chromosomal evolution (Petitpierre and Segarra 1985) based upon 165 species, new findings have been added in the last 25 years (Petitpierre 1999a, 1999b; Petitpierre and Garneria 2003; Gómez-Zurita et al. 2004; Petitpierre and Grobbelaar 2004; Petitpierre et al. 2004; Petitpierre and Elgueta 2006; Petitpierre and Mikhailov 2009), which deserve further approach and discussion in order to improve our views on the cytogenetic evolution and cytotaxonomy of this subfamily.

## Material and methods

The cytogenetic data were mostly obtained by testis dissection of adult or pupa male specimens, which were fixed, teased, squashed, and finally treated by using conventional staining procedures. A great majority of the cells used for these analyses were in meiotic metaphase I stages, which provide the male meioformula, so including the number of autosomal bivalents, male sex-chromosome system, and the possible presence of accessory chromosomes. In addition, less than 50% of the analysed species were also studied in their karyotype architecture from spermatogonial cells in mitotic metaphases or, more seldom, from meiotic metaphase II cells. These more in-depth analyses, gave up worth information on the size and shape of all chromosomes of specific karyotypes, at this second level of cytogenetic resolution known as ß-karyology (White 1978).

Other cytogenetic findings of an even much finer resolution, such as those on genome size, C and/or Ag-banding, and fluorescent *in-situ* hybridization (FISH), have been reported in so few species that they should not be discussed in the frame of this contribution.

Although all the recent and also ancient authors accept the reality of the subfamily Chrysomelinae, the number and names of its tribes and subtribes differ strikingly among them. Thus, very recently, Kippenberg (2010) for instance, proposes five tribes and nine subtribes, based principally on the poorly sampled pupal morphology, whereas Daccordi (1994) and Riley et al. (2002) consider only two tribes, Timarchini and Chrysomelini, the former with one and the latter with four subtribes, and Reid (2002) states four tribes, each one with only one subtribe except Gonioctenini with two. We shall follow here a mixed criterion taken from these latter authors with a few changes according with my own opinions, thereby, we assume the tribes Timarchini and Chrysomelini, the former with the subtribe Timarchina only, and the latter with five subtribes: Entomoscelina (= Phyllocharitina *sensu* Reid 2002), Chrysolinina, Doryphorina, Gonioctenina and Chrysomelina.

## Chromosome numbers and sex-chromosome systems

**Figures 1–3.**
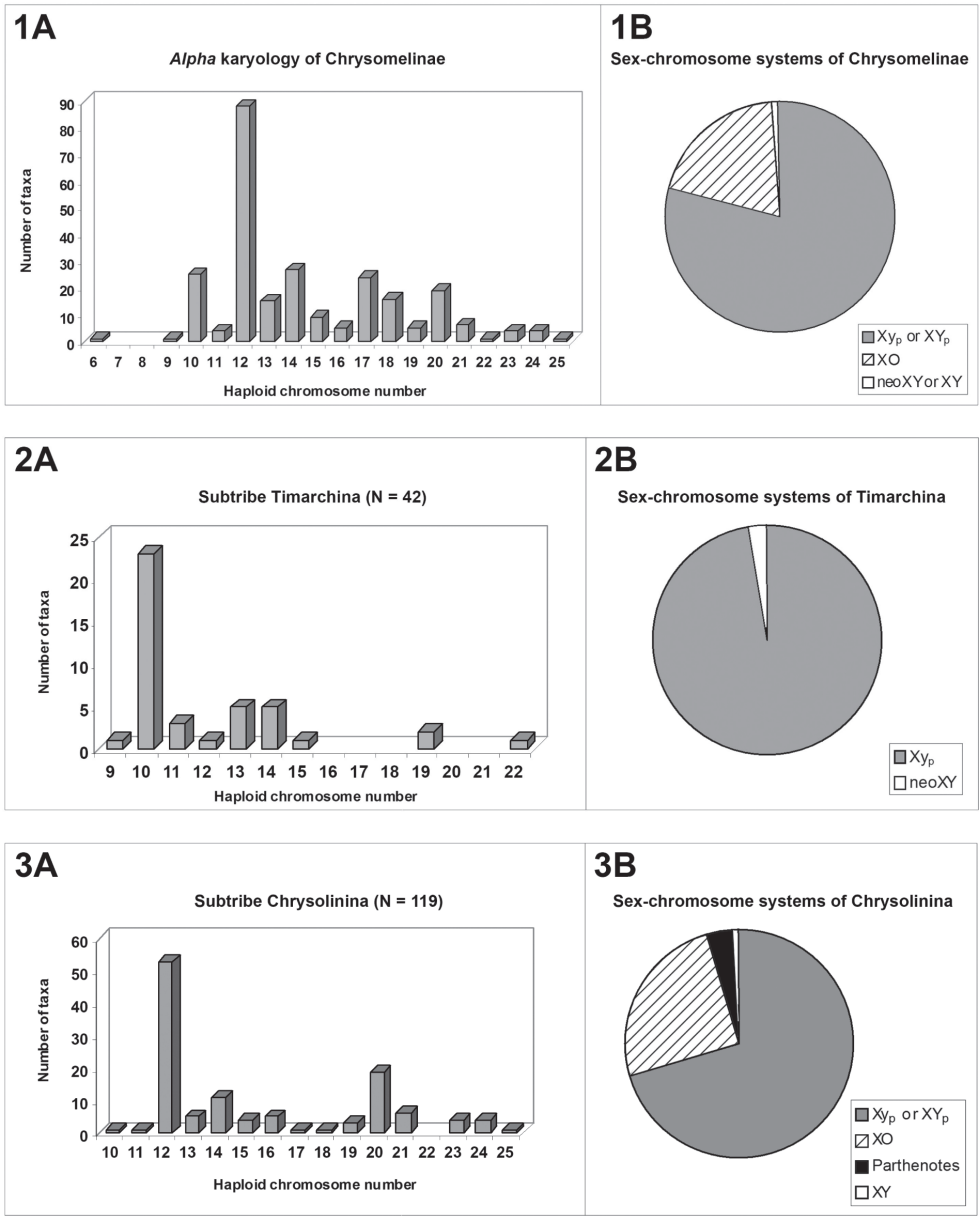
Basic chromosomal data on higher taxa of Chrysomelinae

The Chrysomelinae show a wide variation of diploid chromosome numbers and meioformulas, from 2n = 12 and 5 + neo XY, respectively, in the South American *Doryphora quadrisignata* (Vidal, 1984), to 2n = 50 and 24 + Xy_p_ in the European *Chrysolina rufoaenea* (Petitpierre and Mikhailov, 2009). These shifts in number are almost always due to structural chromosome rearrangements, because only a few polyploidy parthenotes have been recognized to date, all of them restricted to the genus *Calligrapha*, (Robertson 1966, Smith and Virkki 1978). The range of variation of haploid numbers for the total 259 taxa and chromosomal races in the 38 examined genera, shows an almost continuous list of numbers (fig. 1A) but with a modal one of n = 12 (34.2%), followed by three others of n = 10 and n = 17 (both with 9.6%), and n = 20 (7.6%).

Conversely, the parachute-like sex-chromosome system (Xy_p_), of a non-chiasmate nature, is clearly prevailing in the subfamily (79.0%) as shown in fig. 1B. This system consists mostly of a large X and a small y-chromosome, looking such as this configuration at metaphase I, or more rarely, two large X and Y chromosomes (XY_p_), held together by a non-nucleolar argyrophilic substance (Virkki 1984; Postiglioni and Brum-Zorrilla 1988; Virkki et al. 1991). The Xy_p_ is probably the most plesiomorphous condition in Chrysomelinae, as it is for the whole beetles of the suborder Polyphaga (Smith 1951; Smith, 1952; Smith and Virkki, 1978), while the others so far checked in the subfamily, the XO (19.75%) and neoXY or XY systems (1.2%) (fig. 1B), are certainly derived from the former.

**Figures 4–6.**
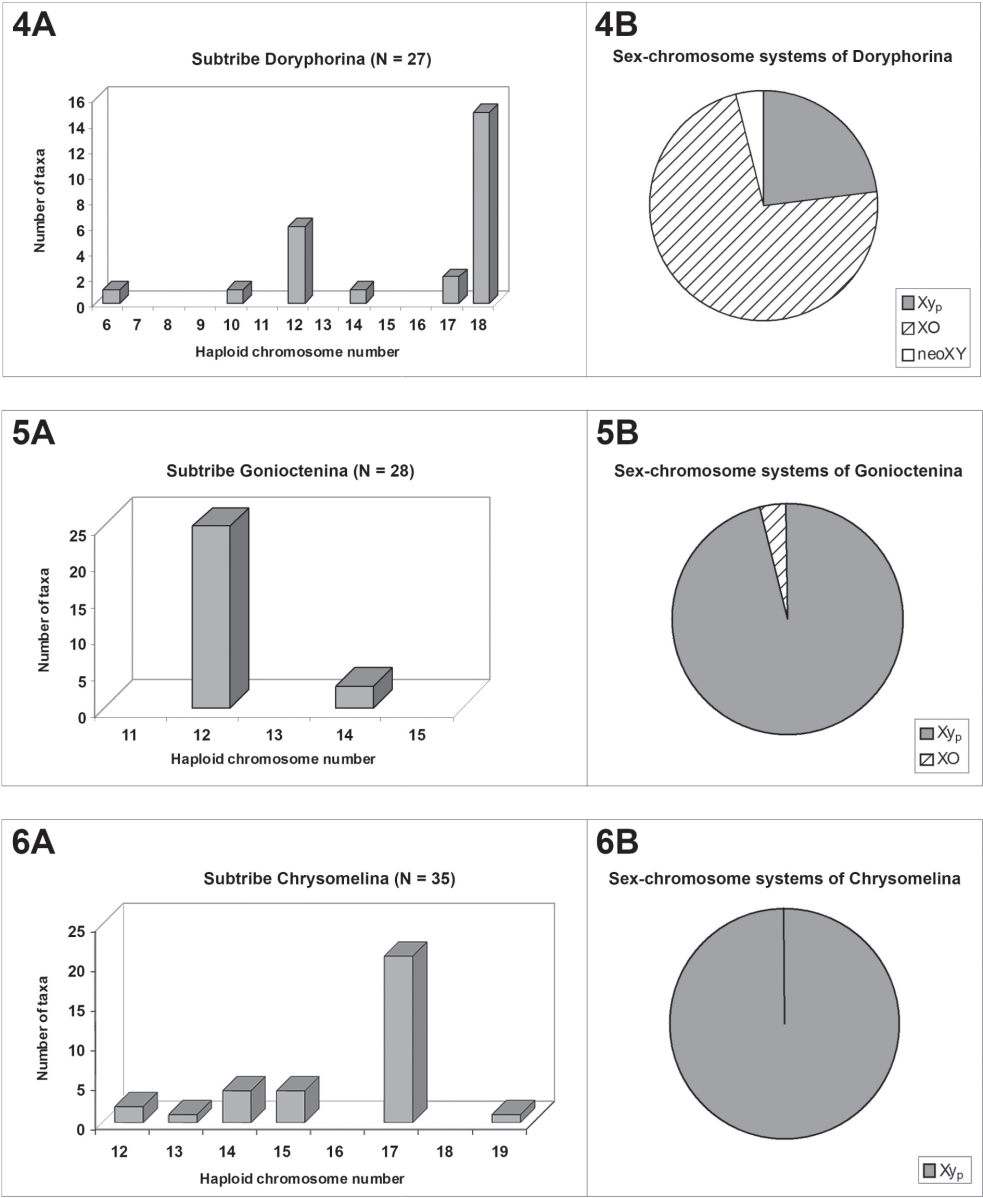
Basic chromosomal data on higher taxa of Chrysomelinae

Although the modal number of n = 12 chromosomes has been found in five out of the six reported subtribes, it is very seldom in Timarchina (fig. 2A) and Chrysomelina (fig. 6A), and it does not occur to date in the poorly surveyed Entomoscelina, with only seven analyzed species (Petitpierre and Grobbelaar 2004, Petitpierre unpublished), belonging to five among the 27 described genera (Daccordi, 1994). Consequently, it can not be presently taken as the presumed ancestral number for the whole subfamily, despite being probably this for the subtribes Chrysolinina (fig. 3A), Gonioctenina (fig. 5A), and less reliably for the Doryphorina (fig. 4A).

The Timarchina subtribe shows a striking modal value of n = 10, and 9 + Xy_p_ meioformula (figs. 2A-2B), which are the modal and presumably the possible plesiomorphous state for this group, as well as for the whole beetles of the suborder Polyphaga (Smith 1952; Smith and Virkki 1978; Angus et al. 2007). However, some caution should be exerted before assuming the previous point, because the two most ancestral extant subgenera of *Timarcha*, *Americanotimarcha* and *Metallotimarc*ha, both on morphological and molecular grounds (Iablokoff-Khnzorian 1966; Jolivet 1989; Gómez-Zurita et al. 2000; Gómez-Zurita 2004), comprise only species showing the highest diploid numbers found in the genus, 2n = 38 and 2n = 44 (Petitpierre and Jolivet, 1976, Jolivet and Petitpierre 1992; Petitpierre, unpublished). If these high numbers were the possible plesiomorphous condition for the chromosomal evolution in *Timarcha*, how could have derived all the common 20-chromosome species by independent processes? The most parsimonious view would be assuming a hypothetic stem species for the genus, represented with a karyotype of 20-chromosomes, from which the ancient ancestors of the three extant subgenera would have splitted. The *Americanotimarcha* and *Metallotimarcha* through multiple chromosome fissions, followed by pericentric inversions and/or chromatin accretions of uniarmed elements, to recover some of them later to their ancient biarmed condition, while within the species-rich *Timarcha* s.str. subgenus much more conservative events of chromosomal shifts had presumably occurred in the karyological origin of most species.

The subtribe Doryphorina displays a 2n(♂) = 35 modal chromosome number and 17 + XO meioformula (figs. 4A–4B), but this can be attributed to a biased sampling on the species of *Leptinotarsa*, all but one sharing these values (Hsiao and Hsiao 1983). Nevertheless, the species of the remaining eight genera of analyzed Doryphorina, out of the two closely related in the genus *Labidomera*, have karyotypes of much lower chromosome numbers, namely, n = 12 in six species of five different genera, *Desmogramma*, *Leucocera*, *Strichosa*, *Platyphora* and *Zygogramma*, a fact which could possibly hint towards the supposed most plesiomorphous karyotype condition for this subtribe too, as we have assumed before.

On the contrary, in subtribe Chrysomelina the modal number and meioformula are 2n = 34 and 16 + Xy_p_, respectively (Figs. 6A and 6B), shared by 65.7% of the 35 surveyed species in twelve genera, and we assumed that this should possibly be the ancestral condition (Petitpierre and Segarra, 1985) for this taxon, but with our present enlarged screening of species and genera, it is more uncertain due to the absence of this 2n = 34(Xy_p_) karyotype and meioformula in half of the twelve sampled genera.

If we study the α-karyology of chrysomelines at the genus level, we find genera with high chromosomal diversity, as measured by standard deviation (SD) of their male diploid chromosome numbers, for example *Chrysolina* with SD = 8.67 in 72 sampled taxa and chromosomal races, *Timarcha* with SD = 4.33 in 42 taxa, and *Cyrtonus* with SD = 6.33 in 15 taxa, whereas other genera have zero or a low diversity such as *Paropsisterna* with SD = 0 in 10 taxa, *Chrysomela* with SD = 0 in 9 taxa, *Oreina* with SD = 1.15 in 12 taxa, and *Leptinotarsa* with SD = 2.77 in 16 taxa. The differences between “variable” and “conservative” genera in their chromosome numbers, were tentatively explained according with the ability for dispersal of flying vs. flightless species genera, and the number of host-plant families they are able to feed, being both characters in a presumed relationship with the size of local populations and thereby with the chances of fixation for new chromosomal shifts (Petitpierre et al. 1993). Under these premises, the genera with flying species and feeding on only one or two plant-families would presumably constitute larger local populations and, consequently, they are less able to fix new chromosomal mutations by random genetic drift and/or inbreeding than those genera of flightless species and feeding on a good number of plant families as particular habitats to live and breed for each beetle species. *Timarcha* and *Cyrtonus* consist of apterous species only, most *Chrysolina* have wings but are flightless, and these three genera feed on six, one, and seven plant families respectively, and they share high heterogeneities of diploid numbers (SD > 4.0); on the contrary, *Paropsisterna*, *Chrysomela* and *Leptinotarsa* have flying species, the first two feeding on a unique plant family each and the third on three, but *Oreina* has some species completely unable to fly and others flying very seldom, in spite of being chromosomally “conservative” as the previous three genera (SD ≤ 4.0), and feeding on two plant families only (Petitpierre et al. 1993). Hence, these two features alone can not account for all the observed intrageneric variation in diploid chromosome numbers of these chrysomelines.

## Evolution of chromosomal architecture

The chromosomes may show a huge variable morphology in size and shape, some species have karyotypes made of very few chromosomes of a large size while others have karyotypes of many small chromosomes and there are not evidences of any advantages of ones over others, although minute chromosomes are more easily lost at meiosis if a chiasma fails to be formed, and very large acro- or telocentric chromosomes can be cut across before they have been properly separated at anaphase (Sumner 2003). Anyway, chromosomes are elements of the genetic system that may supply worth features to explain evolution among closely related species (White 1973, King 1993).

Some 80 among the 259 presently know taxa or chromosomal races of chrysomelines have been examined at the level of ß-karyology i.e. by identifying size and shape of individual chromosomes in each karyotype. Such kind of studies have been mainly carried out in certain genera, the North American *Calligrapha* (Robertson 1966) and *Leptinotarsa* (Hsiao and Hsiao 1983), and the Holarctic *Timarcha* (Petitpierre 1970, 1976; Gómez-Zurita et al. 2004), and the Palaearctic *Chrysolina* (Petitpierre 1981, 1983, 1999a, 1999b; Petitpierre et al. 2004; Petitpierre and Mikhailov 2009) and *Cyrtonus* (Petitpierre and Garneria 2003). The karyotypes of chrysomelines are usually composed of meta- or submetacentric chromosomes as occur mostly in all groups of Coleoptera (Smith and Virkki 1978; Virkki 1984). This means that the shifts in number due to centric fissions, should necessarily rebuild the emerging acrocentric chromosomes into biarmed ones by pericentric inversions or heterochromatin accretions (Virkki 1984; Virkki and Santiago-Blay 1993), and this secondary metacentry has been described in diphasic chromosomes of several beetle species (Virkki 1984). Taking into account the biarmed shape of most chromosomes in chrysomelines, and in other beetles in general, it is evident that the number of major chromosome arms (FN = fundamental number) could not remain constant and increase accordingly with the diploid number. Nevertheless, many species of high diploid numbers have at least a few acrocentric or subacrocentric chromosomes, which may be the ancient survivors of primary shifts by centric fissions. For instance, the Nearctic *Timarcha intricata* with 2n = 44 has 15 of such autosome pairs (Petitpierre and Jolivet 1976; Petitpierre 1988), *Leptinotarsa lineolata*, *Leptinotarsa behrensi* and *Leptinotarsa decemlineata* (the potato beetle), all with 2n (♂) = 35, have seven, four and three, respectively (Hsiao and Hsiao 1983), the Palaearctics *Timarcha pimelioides* with 2n = 28 has five (Petitpierre 1976, 1988), *Chrysolina gypsophilae* with 2n = 32 has three (Petitpierre 1999b), *Chrysolina diluta* with 2n = 36 and *Chrysolina haemoptera* with 2n = 40 have four (Petitpierre 1988), *Chrysolina lepida* with 2n = 42 has six, whereas its closely related *Chrysolina fuliginosa*, also with 2n = 42, has none (Petitpierre 1999a). The extreme cases are those of the European *Chrysolina carnifex* and *Chrysolina interstincta* both with 2n = 40 and having only acrocentric chromosomes, contrary to *Chrysolina helopioides* with 2n (♂) = 47 and lacking any of them (Petitpierre 1981; Petitpierre 1999a; Petitpierre and Segarra 1985; Petitpierre et al. 2004). In conclusion, the FN even in species having similar numbers as the latter, can be strikingly distinct, FN = 40 in *Chrysolina carnifex* and *Chrysolina interstincta*, and FN = 94 in *Chrysolina helopioides*. Additional examples of frequent increases of acrocentric chromosomes in Polyphaga beetles associated with high diploid numbers are those which have been reported in Buprestidae (Karagyan and Lachowska 2007) and in Curculionidae (Lachowska et al. 1998).

Karyotypes can also be classified as symmetrical in size when all chromosomes have similar magnitudes, and asymmetrical when there are two clearly distinct size classes, and these two alternatives can also be applied to chromosome shape, uniarmed chromosomes for asymmetrical and biarmed ones for symmetrical karyotypes (Stebbins 1971; White 1973).

For the sake of simplicity we should only consider here the asymmetry vs. symmetry in chromosome size but not in shape. The karyotypes of Chrysomelinae offer examples of both types but more often of intermediate states, that is, with chromosomes of gradually decreasing sizes. In order to measure the degree of asymmetry of a karyotype we have used the standard deviation (SD) of each chromosome relative length with respect to the averaged % length taken from the total complement length (TCL) (Petitpierre and Segarra 1985). Here again we use this parameter but measuring the % of each chromosome length at mitotic metaphase with regard to the haploid TCL including the X but not the Y-chromosome, therefore, treating identically the species with or without a Y-chromosome. In this sense, we have calculated the SDs of asymmetry in 63 species and subspecies, whose karyotypes were mostly published, from the following ten genera of chrysomelines: the Holarctic *Timarcha* (Petitpierrre 1970, 1976), and the Nearctics or Palaearctics *Calligrapha* (Robertson 1966), *Chrysolina* (Petitpierre 1983, 1999a, 1999b; Petitpierre and Segarra 1985; Petitpierre et al. 2004), *Oreina* (Petitpierre 1999a), *Cyrtonus* (Petitpierre and Segarra 1985; Petitpierre and Garneria 2003), *Leptinotarsa* and *Labidomera* (Hsiao and Hsiao 1983), *Phratora* (Petitpierre and Segarra 1985), and the Neotropical *Araucanomela* (Petitpierre and Elgueta 2006) and *Henicotherus* (Petitpierre unpublished).

These cytogenetic results are reported in Table 1 and they were used to obtain the coefficient of correlation (r) between these two cytological parameters, haploid chromosome number and SD of karyotype asymmetry, which was clearly negative with a highly significant likelihood, r = - 0.74 (P > 0.99). In brief, the increase in haploid chromosome number is generally associated with a decrease in asymmetry, or in other words, the karyotypes are more symmetrical when they have more chromosomes, a clear trend which has also been reported in other beetles like the weevils (Curculionidae) by Lachowska et al. (1998). This does not mean at all an evident polarity towards increases in chromosome number by centric fissions, although it seems to be the more feasible trend in leaf beetles (Petitpierre and Segarra 1985; Virkki 1970, 1988; De Julio et al. 2010). Nevertheless, some well-established examples in chrysomelines support the reverse shifts in number by centric fusions: a) the origin of chiasmatic sex-chromosome systems neo-XY from the non-chiasmatic Xy_p_ or XY_p_ imply a translocation between an autosome and the X-chromosome, with the loss or fusion of the y-chromosome. The karyotype with the lowest number reported to date in chrysomelines, that of *Doryphora quadrisignata*, with 5 + neo XY meioformula (Vidal 1984), has probably arisen by a centric fusion of this previous type plus several further fusions between autosomes, b) the meioformula of *Timarcha aurichalcea*, 8 + neoXY, the lowest one so far found in this genus, has been clearly demonstrated to be due to an all-arm translocation between a X-chromosome and one autosome bearing the rDNA loci, by fluorescent *in situ* hybridization (FISH) using a ribosomal DNA probe (Gómez-Zurita et al. 2004), and c) the origin of the strikingly asymmetric karyotype of *Chrysolina* (*Stichoptera*) *kuesteri* with 2n = 22 chromosomes (Petitpierre 1983), can be presumably explained from a 24-chromosome species of the same subgenus, such as *Chrysolina latecincta*, because the largest autosome of the former has 21.30% of the complement length while that of the latter has 16.46% only, therefore, a centric fusion between this largest autosome and a smaller one of *Chrysolina latecincta*, or any other karyologically similar species of the subgenus *Stichoptera*, may have given rise after fixation to the largest autosome pair of *Chrysolina kuesteri* (Petitpierre, 1999b).

**Table 1.** Haploid chromosome number (n) and SD of karyotype asymmetry

	**n**	**DS**		**n**	**DS**
*Timarcha balearica*	11	3.57	*Cyrtonus cobosi*	14	3.36
*Timarcha calceata*	15	2.30	*Cyrtonus contractus*	14	2.19
*Timarcha cyanescens*	10	4.71	*Cyrtonus elegans*	14	2.11
*Timarcha erosa vermiculata*	10	6.28	*Cyrtonus plumbeus*	14	2.29
*Timarcha fallax*	10	4.66	*Oreina ludovicae*	12	3.32
*Timarcha intermedia*	10	3.41	*Calligrapha alni*	12	3.73
*Timarcha lugens*	10	4.02	*Calligrapha amator*	12	3.42
*Timarcha marginicollis*	10	4.09	*Calligrapha bidenticola*	12	3.32
*Timarcha pimelioides*	14	4.01	*Calligrapha californica corepsivora*	12	4.76
*Timarcha recticollis*	10	4.72	*Calligrapha confluens*	12	3.32
*Timarcha rugosa*	13	3.45	*Calligrapha multipunctata bigsbyana*	12	3.39
*Timarcha sicelidis*	10	5.27	*Calligrapha philadelphica*	12	3.14
*Timarcha strangulata*	14	1.68	*Calligrapha pnirsa*	12	3.41
*Chrysolina affinis baetica*	12	1.65	*Calligrapha pruni*	12	3.95
*Chrysolina americana*	12	1.98	*Calligrapha rowena*	12	3.42
*Chrysolina bankii*	12	2.12	*Calligrapha verrucosa*	12	3.16
*Chrysolina bicolor*	12	1.60	*Labidomera clivicollis*	17	2.36
*Chrysolina carnifex*	20	1.29	*Labidomera suturella*	16	2.11
*Chrysolina coerulans*	12	3.05	*Leptinotarsa behrensi*	18	1.84
*Chrysolina costalis*	12	2.54	*Leptinotarsa decemlineata*	18	1.51
*Chrysolina femoralis*	12	1.65	*Leptinotarsa defecta*	18	1.83
*Chrysolina gypsophilae*	16	2.76	*Leptinotarsa haldemani*	18	1.38
*Chrysolina haemoptera*	20	1.54	*Leptinotarsa heydeni*	18	1.52
*Chrysolina helopioides*	24	1.51	*Leptinotarsa juncta*	18	1.20
*Chrysolina herbacea*	12	2.56	*Leptinotarsa lineolata*	18	1.62
*Chrysolina hyperici*	19	1.37	*Leptinotarsa peninsularis*	18	1.83
*Chrysolina kuesteri*	11	5.41	*Leptinotarsa rubiginosa*	18	1.36
*Chrysolina latecincta*	12	4.74	*Leptinotarsa texana*	18	1.45
*Chrysolina umbratilis*	15	3.08	*Leptinotarsa tumamoca*	18	1.55
*Phratora tibialis*	17	1.63	*Leptinotarsa typographica*	18	1.48
*Henicotherus porteri*	14	2.14	*Leptinotarsa undecimlineata*	17	2.67
			*Araucanomela wellingtonensis*	14	4.49
